# Artemisinin-Resistant Malaria: Research Challenges, Opportunities, and Public Health Implications

**DOI:** 10.4269/ajtmh.2012.12-0025

**Published:** 2012-08-01

**Authors:** Rick M. Fairhurst, Gaurvika M. L. Nayyar, Joel G. Breman, Rachel Hallett, Jonathan L. Vennerstrom, Socheat Duong, Pascal Ringwald, Thomas E. Wellems, Christopher V. Plowe, Arjen M. Dondorp

**Affiliations:** Laboratory of Malaria and Vector Research, National Institute of Allergy and Infectious Diseases, National Institutes of Health, Bethesda, Maryland; Fogarty International Center, National Institutes of Health, Bethesda, Maryland; Faculty of Infectious and Tropical Diseases, London School of Hygiene and Tropical Medicine, London, United Kingdom; College of Pharmacy, University of Nebraska Medical Center, Omaha, Nebraska; National Center for Parasitology, Entomology and Malaria Control, Phnom Penh, Cambodia; Global Malaria Programme, World Health Organization, Geneva, Switzerland; Howard Hughes Medical Institute and Center for Vaccine Development, University of Maryland School of Medicine, Baltimore, Maryland; Mahidol-Oxford Tropical Medicine Research Unit, Faculty of Tropical Medicine, Mahidol University, Bangkok, Thailand

## Abstract

Artemisinin-based combination therapies are the most effective drugs to treat *Plasmodium falciparum* malaria. Reduced sensitivity to artemisinin monotherapy, coupled with the emergence of parasite resistance to all partner drugs, threaten to place millions of patients at risk of inadequate treatment of malaria. Recognizing the significance and immediacy of this possibility, the Fogarty International Center and the National Institute of Allergy and Infectious Diseases of the U.S. National Institutes of Health convened a conference in November 2010 to bring together the diverse array of stakeholders responding to the growing threat of artemisinin resistance, including scientists from malarious countries in peril. This conference encouraged and enabled experts to share their recent unpublished data from studies that may improve our understanding of artemisinin resistance. Conference sessions addressed research priorities to forestall artemisinin resistance and fostered collaborations between field- and laboratory-based researchers and international programs, with the aim of translating new scientific evidence into public health solutions. Inspired by this conference, this review summarizes novel findings and perspectives on artemisinin resistance, approaches for translating research data into relevant public health information, and opportunities for interdisciplinary collaboration to combat artemisinin resistance.

## Introduction

Communities combating malaria have new grounds for optimism, as 40% of the world's malaria-endemic countries reported their cases dropping by half over the last decade.^1^ These recent successes, accompanied by a 15-fold increase in funding over the last decade, have fueled global interest in malaria and shifted the world's focus from controlling to eliminating this dreadful disease.^1^,^2^ Past experiences have shown that reductions in malaria incidence can be achieved but are difficult to sustain. Parasite resistance to antimalarial drugs and mosquito resistance to insecticides both contributed significantly to the failure of a worldwide campaign to eradicate malaria in the latter half of the last century^3^; today, drug-resistant malaria threatens the global community's major recent investment in rolling out effective new drug combinations to replace failed and failing older drugs.

*Plasmodium falciparum*, which causes the most life-threatening malaria syndromes, has developed resistance to almost every class of antimalarial compounds.^4^ Regional burdens of falciparum malaria can surge dramatically when parasites become resistant to commonly used drugs. For instance, child mortality from falciparum malaria increased significantly in the 1980s as chloroquine-resistant parasites arrived in sub-Saharan Africa and spread across the continent.^5^–^6^ After *P. falciparum* developed resistance not only to chloroquine, but also to sulfadoxine-pyrimethamine, mefloquine, and other antimalarial drugs, the World Health Organization (WHO) recommended artemisinin-based combination therapy (ACT) as first-line treatment of falciparum malaria. ACT is the combination of artemisinin or an artemisinin derivative (e.g., artesunate, artemether, dihydroartemisinin) and a partner drug (e.g., amodiaquine, mefloquine, piperaquine, lumefantrine) having a markedly longer half-life in the bloodstream than artemisinin.

One rationale for ACT is that the highly potent artemisinins have a rapid onset of action that is accompanied by a very short half-life. Thus, although a 3-day regimen of artemisinin precipitously reduces the parasite biomass, the longer-acting but less potent partner drug is required to kill any remaining parasites over 1–2 weeks. Importantly, in addition to its benefits in the treatment of uncomplicated malaria, artesunate is also more effective than quinine in reducing the mortality of severe falciparum malaria in Asian adults and in sub-Saharan African children.^7^,^8^ To avoid losing the potency and live-saving capacity of artemisinins to the development of resistance, researchers have recommended for years that artemisinins be used in combined regimens for uncomplicated malaria. Unfortunately, the use of artemisinin monotherapy for treatment of uncomplicated malaria continues to be a common practice in malaria-endemic areas.^9^

In 2005, a WHO report on reduced susceptibility of *P. falciparum* to antimalarial drugs warned against the possibility and danger of artemisinin resistance^10^; in the following year, the WHO recommended that artemisinin monotherapy be eliminated altogether. Although some countries have enforced the ban,^9^ many pharmaceutical companies continue to produce and distribute a variety of artemisinin monotherapies to malarious countries with few regulatory obstacles.^11^ As had been feared, impaired parasite responses to artemisinin monotherapy eventually emerged and are now well-established in the Cambodia-Thailand border region, a historical epicenter for the development and spread of antimalarial drug resistance.^12^,^13^ Reduced sensitivity to artemisinins in turn renders the ACT partner drugs more vulnerable to the development of resistance. This ominous development, along with the previous emergence of parasite resistance to all currently used partner drugs, forecasts that current ACT regimens will begin to fail. This will lead to recurrent malaria after treatment of initial malaria episodes, to compromised efficacy of artemisinin-based treatment of severe malaria, and to failure of ACTs to eradicate parasites from infected individuals in malaria control and elimination programs.

Recognizing the significance and immediacy of these challenges, The Fogarty International Center and the National Institute of Allergy and Infectious Diseases of the U.S. National Institutes of Health convened a conference in November 2010 to bring together the diverse array of stakeholders affected by the growing threat of artemisinin resistance, including scientists and public health officials who work in malarious countries at peril. The objectives of the conference were to 1) review the status of artemisinin resistance and the implications of this finding for malaria control and elimination efforts; 2) to identify actions and recommendations to slow the spread of resistance and the emergence of new foci for policymakers, public health officials, regulatory agencies and donors; 3) to highlight opportunities and knowledge gaps where scientific discoveries can mitigate or eliminate present challenges; and 4) to catalyze and intensify collaborations between field- and laboratory-based researchers and health workers, with the aim of accelerating the translation of new scientific findings to the field in an effort to forestall the development of artemisinin resistance and avoid its deleterious consequences on the world's most vulnerable populations.

Several research groups working in Southeast Asia have played key roles in detecting the first signs of artemisinin resistance, which threatens to compromise the efficacy of all ACTs. These groups are now working to define the geographic distribution and intensity of this phenomenon through *in vivo*, *in vitro*, and molecular studies. The earliest of these joint efforts was the “Artemisinin Resistance Confirmation, Characterization and Containment” (ARC3) project, coordinated in Southeast Asia by WHO starting in 2008, followed by a workshop and a second round of studies to develop a strategy for containment of artemisinin resistance, the Artemisinin Resistance Containment (ARCE) project, also coordinated by the WHO. More recently, the Tracking Resistance to Artemisinins Collaboration (TRAC) was established to better define and map parasite resistance to artemisinin-based therapies in a series of studies across Southeast Asia and Africa. With so many gaps in our understanding of how artemisinin resistance has emerged in the Cambodia-Thailand border region and how it might spread or independently arise in other areas, including Africa, integrated programs of basic and clinical research like ARC3, ARCE, and TRAC will be crucial to successfully address this public health emergency.^14^ Joint efforts of research groups, policy makers, and funders will continue to be needed to forestall widespread ACT failure and to search for new antimalarial drugs.^15^ This report summarizes some of the most salient points presented at the conference and discussed among participants over the past year.

### Defining artemisinin resistance: A work in progress.

Defining artemisinin resistance is a work in progress and currently no consensus exists on a standard definition; thus, claims of artemisinin resistance should be considered with caution.

#### In vivo studies.

Defining artemisinin resistance in human populations remains a challenge for clinicians, scientists, and policy makers. The WHO's working definition “suspects” or “confirms” artemisinin resistance based on clinical and parasitological outcomes observed during routine therapeutic efficacy studies of ACTs or clinical trials of artesunate monotherapy, respectively.

##### Suspected resistance.

When standard oral regimens of an ACT are directly administered over 3 consecutive days, artemisinin resistance is suspected when ≥ 10% of patients still have parasitemia at least 72 hours after initiation of treatment (detected by examining a thick blood film for asexual-stage *P. falciparum* parasites). The 72-hour threshold is presently used because a recent meta-analysis of parasite clearance times^12^,^16^ showed that parasites cleared in ≤ 72 hours in 97% of patients if initial parasite density is between 10,000 and 100,000/μL of whole blood.^16^

Because this definition of suspected artemisinin resistance is based on a single 72-hour test result, it has important limitations. First, parasite clearance at 72 hours depends on the initial parasite density. This is because each of the three doses of artemisinin in an ACT produces a 10^4^-fold reduction in parasite density during each asexual-stage development cycle. Therefore, three doses of ACT may not be sufficient to reduce very high parasite densities (> 100,000/μL of whole blood) to zero by 72 hours. Second, the definition may have limited application in some areas where the levels of acquired immunity are much higher than in Southeast Asia. In such settings, parasite clearance may be relatively fast,^16^ and a delay in parasite clearance from 24 to 48 hours over time may indicate a decline in parasite susceptibility to artemisinin but not meet the WHO definition of delayed clearance > 72 hours. Third, because the definition relies on data obtained from a single 72-hour time point, the microscopist's skill in detecting parasitemia on blood films prepared under field conditions becomes critical. Unfortunately, this type of surveillance activity is currently not available in much of sub-Saharan Africa and would require significant investment in establishing and monitoring sentinel sites. Fourth, because the ACT partner drug also contributes to parasite clearance, declining efficacy of this drug may delay parasite clearance. In light of this, concurrent evaluation of the partner drug's efficacy may be warranted in some areas. Fifth, we do not yet know whether the various artemisinin derivatives in ACTs vary significantly in their ability to clear parasites during the first 72 hours of treatment. Finally, 72-hour positivity rates may be overestimated if blood smears are made at more convenient time points, rather than more accurately at 72 hours after the first dose of artemisinin is given. In addition, other parameters, including red cell phenotypes, drug quality, and pharmacokinetic profiles can differ between patients and may affect parasite clearance.

##### Confirmed resistance.

When standard oral regimens of artemisinin monotherapy (2–4 mg/kg/day) are administered over 7 consecutive days, and adequate levels of drug are documented in plasma, the WHO confirms resistance if parasites are still present on Day 7 (i.e., at 168 hours), or if parasites are present at 72 hours and then recrudesce (i.e., initially clear but then reappear as late as day 42).^17^ In most settings, it is not practical to hospitalize patients in mosquito-free environments for 42 days; therefore, parasites that reappear after initially clearing must be genotyped to confirm that any recrudescent clone was present at the time of initial treatment. Furthermore, recrudescent infections are not uncommon after 7 days of treatment with artemisinin monotherapy, even in the absence of artemisinin resistance, with recrudescence rates around 11% at Day 42.^18^

The WHO recommends that containment activities begin even when resistance is only suspected, whereas more intensive studies are conducted to confirm artemisinin resistance. These confirmatory studies are centered on estimating the parasite clearance rate^19^,^20^ while a patient completes a 7-day regimen of artemisinin alone, or a staggered 5- to 6-day regimen of ACT in which the artemisinin component is taken for the first 3 days (Days 0, 1, and 2), followed by the partner drug for the next 2 or 3 days or, as more recently used in the TRAC studies, a full ACT regimen starting on Day 3 (i.e., at 72 hours). These dosing regimens enable investigators to attribute the parasite clearance rate over the first 72 hours to artemisinin itself and not the partner drug. Because, in the absence of resistance, artemisinin results in rapid parasite clearance, the assessment of a patient's blood parasite density every 6 hours until undetectable is an adequately sensitive measurement. From the log_e_-linear portion of the parasite clearance curve, a slope can be calculated. Although there is presently no slope cutoff for defining artemisinin resistance, a slowing of the parasite clearance rate over time in any given region would be highly suggestive.

A potential problem with this approach to defining artemisinin resistance is that the parasite clearance rate may be affected by factors other than the intrinsic susceptibility of a parasite isolate to artemisinin *in vivo*, or splenic function.^21^ Other host factors have been postulated to affect the parasite clearance rate. The effect of hemoglobin E, a hemoglobin variant carried by up to 50% of Khmer individuals living in Western Cambodia, on parasite clearance has not been established. Innate and adaptive immune responses likely promote parasite clearance, but their contribution to parasite clearance rates in Southeast Asia is difficult to quantify. This is because age is not an adequate surrogate for levels of adaptive immunity (unlike in Sub-Saharan Africa) and robust *in vitro* correlates of innate and adaptive immunity have not yet been identified in Southeast Asian study populations. One tool to investigate the relative contributions of host and parasite factors in clearance dynamics is the genotyping of parasite isolates showing a variety of clearance rates^13^; in low transmission settings, genotyping results may reveal that highly-related parasite clones are present in different patients. The degree of clustering of parasite clearance rates (fast or slow) in patients infected with highly-related parasite clones is a measure of the parasite “heritability” of the clearance phenotype. The “heritability” not attributed to parasite genetics may be caused by host genetics or other factors that have not yet been identified.

The potential to use measures of post-treatment gametocytemia as *in vivo* correlates of early-stage artemisinin resistance is presently under investigation. The rationale for this approach comes from multiple studies of treatment with older antimalarials, in particular chloroquine and sulfadoxine-pyrimethamine, which showed higher prevalence and density of gametocytes with resistant genotypes after treatment. Of importance, the association was measurable before clinical efficacy had waned^22^–^24^; one potential mechanism for this phenomenon is that partially effective drugs are unable to clear minor populations of resistant asexual-stage parasites and immature gametocytes. Seven to 10 days after treatment, the pool of circulating mature gametocytes is then enriched by those that survived drug treatment at an earlier developmental stage. Where membrane-feeding assays have been carried out, it was also found that increased post-treatment gametocytemia translated into enhanced transmission of drug-resistant parasites from patients to mosquitoes.^25^,^26^

At present, it is not known whether slow clearance of artemisinin-treated parasites is associated with increases in gametocyte prevalence, density, or infectivity to mosquitoes. Recently, however, one study from the Thailand-Myanmar border has reported that in patients treated with artesunate-mefloquine, delayed parasite clearance was associated with increased risk of developing gametocytemia.^27^ If such increases are found to be attributable to the artemisinin component of ACTs, then post-treatment quantification of gametocytes could be useful in surveillance for artemisinin resistance and would suggest increased transmissibility of artemisinin-resistant parasites. Initial screening studies may only require thick blood smears on Days 7 and 14 after treatment. This could enable investigators to determine whether sequestered immature gametocytes (which can be detected on Day 7 as circulating mature gametocytes) or very early ring forms (which take 14 days to commit and develop into circulating mature gametocytes) are developing tolerance to artemisinins.

#### In vitro studies.

A variety of conventional *in vitro* tests provide a quantitative measure of the intrinsic susceptibility of *P. falciparum* parasites to antimalarial drugs. This is usually expressed as the IC_50_, the concentration of drug that inhibits parasite growth by 50% (as determined by quantifying parasite DNA replication by radioisotopic methods, monitoring the maturation of ring to schizont stage parasites by microscopy or flow cytometry, counting new ring-stage parasites after one cycle of parasite growth and re-invasion, or quantifying the levels of parasite-specific proteins).^28^–^30^ Some of these standard *in vitro* assays have produced IC_50_ values showing weak or no correlations with the *in vivo* phenotype of parasite clearance rate,^12^,^14^ suggesting the need for a different type of *in vitro* test. Research to develop new *in vitro* assays to distinguish fast- from slow-clearing *P. falciparum* isolates is underway. Modeling methods suggest that the artemisinin resistance phenotype is associated with the immature ring stages of parasite development, as opposed to the mature trophozoite and schizont stages.^31^ *In vitro* tests that focus on the inhibition of ring-stage parasites growth, support this finding.^32^–^34^ If validated as a correlate of delayed clearance *in vivo*, standardized, high-throughput *in vitro* methods for measuring refractoriness of ring forms to artemisinin could be a valuable surveillance tool.

The mechanism by which artemisinins kill *P. falciparum* has not been firmly established. Identifying the mechanism of drug action will likely improve our understanding of how parasites are becoming more tolerant to artemisinins and enable us to develop an *in vitro* assay that effectively measures artemisinin resistance. There is a growing body of published and unpublished data on the response of *P. falciparum* parasites to artemisinin.^34^ When *P. falciparum* is exposed to artemisinin *in vitro*, a fraction of ring-stage parasites seems to enter a dormant state.^32^ This has been demonstrated by pulsing *P. falciparum* with artemisinin at doses that seemingly kill all parasites (only pyknotic forms remain), washing away the drug, and then cultivating the parasites for up to several weeks. At particular doses and durations of artemisinin exposure, a fraction of parasites survive and eventually replicate to levels that can be detected in blood smears or by flow cytometric methods. *P. falciparum* lines that resist artemisinin exposure may therefore include a higher fraction of slow-clearing parasites that revive from dormancy compared with fast-clearing parasites.

#### Molecular marker studies.

Molecular markers are particularly useful for large-scale surveillance studies, as they can be more easily standardized and rapidly deployed than *in vivo* and *in vitro* methods for monitoring resistance.^35^ In addition, identification of molecular markers will reveal insights into the mechanisms of artemisinin drug action and resistance. Much of the research to identify markers of artemisinin resistance has followed a candidate gene approach, focusing on genes known to play a role in parasite resistance to other drugs or genes involved with mechanisms of drug action (Table 1). Other research efforts have investigated changes in the expression of parasite genes on exposure to high doses of artesunate. By this approach, for example, one study has implicated increased expression in P-Art, a novel protein of unknown function, in decreased parasite susceptibility to artesunate *in vitro*.^52^,^53^ Although *in vitro* evidence has suggested that some candidate gene variants may play a modulatory role in parasite susceptibility to artemisinins, no correlations between the presence of these variants and parasite clearance *in vivo* was observed in Western Cambodia.^54^

A comprehensive genome-wide search, as a complementary approach to other strategies, may help to identify the parasite genetic determinants of artemisinin resistance. Although genome-wide association (GWA) studies identified genetic loci associated with *in vitro* susceptibility of *P. falciparum* to artemisinins,^55^–^57^ these loci have not yet been implicated in slow parasite clearance in patients with malaria. A recent study found that highly related parasite clones in multiple patients in Western Cambodia share correspondingly fast or slow parasite clearance rates, suggesting a parasite genetic contribution to artemisinin resistance.^13^ These data suggest that the parasite clearance rate is a suitable phenotype for GWA and other genomic studies, which may eventually identify candidate resistance markers to be validated and used in future surveillance efforts. These include standard and exploratory bioinformatics analyses of whole parasite genome sequences.

### Mechanisms of artemisinin drug action, and the need for new antimalarial drugs effective against artemisinin-resistant *P. falciparum.*

Considerable evidence suggests that the pharmacophoric peroxide bond in artemisinin undergoes reductive activation by heme released by hemoglobin digestion in the parasite digestive vacuole.^58^–^60^ This irreversible redox reaction produces carbon-centered radicals or carbocations that alkylate heme^61^ leading to oxidation reactions that damage parasite membranes.^62^ Indeed, the activity of artemisinin is dependent upon hemoglobin digestion,^63^ consistent with the specificity and efficacy of this drug against hemoglobin-degrading pathogens.^64^ Alternatively, it has been postulated that artemisinin oxidizes parasite FADH_2_ and parasite redox-active flavoenzymes^65^ or undergoes reductive activation in parasite mitochondria,^66^ both of which are thought to cause parasite death by an increase in reactive oxygen species. Yet another possible mechanism is that artemisinin inhibits PfATP6, a *P. falciparum* sarcoendoplasmic reticulum Ca^2+^-ATPase (SERCA) homolog.^67^ While one study found a correlation between PfATP6 polymorphism and reduced *in vitro* susceptibility to artemisinins of *P. falciparum* isolates from French Guiana,^68^ this correlation was not observed in another study.^62^ Artemisinin metabolites have also been found to alkylate-specific *P. falciparum* proteins, including a translationally controlled tumor protein homolog.^69^ Although these studies have investigated different mechanisms of artemisinin action, it will be important to provide conclusive evidence for them and to synthesize the available data into a model of drug action that operates *in vivo*.

Elucidating the mechanism of artemisinin action with greater clarity may improve our understanding of artemisinin resistance. The key role of hemoglobin digestion in the mechanism of action of artemisinin^63^ is consistent with one hypothesis that the artemisinin resistance phenotype is associated with the developmental arrest of immature or early ring stages of the parasite in which hemoglobin digestion has only just begun in pre-digestive vacuole compartments.^70^,^71^ Other mechanisms that enable parasites to better tolerate the effects of artemisinin could involve mutations, amplifications, or altered expression of genes encoding drug targets, drug transporters, or enzymes and metabolites involved in antagonizing drug action. Identifying these molecules could facilitate the discovery of new antimalarial drugs that target these same molecules or other constituents of the biochemical pathways involved. Promising new compounds may be able to prevent *P. falciparum* from entering a quiescent stage altogether, or greatly prolong the time at which parasites recrudesce *in vitro* after artemisinin exposure. A recent study showed that simply increasing the daily dose of artemisinin to 6 or 8 mg/kg from 4 mg/kg is not an effective treatment option, because this dose range does not accelerate parasite clearance and is associated with transient neutropenia.^72^ This finding highlights the importance of identifying antimalarial compounds with novel mechanisms of action that can effect similarly dramatic reductions in parasite densities.

The identification of new artemisinin partner drugs for next generation ACTs is similarly critical, not only to counter emerging resistance to existing partner drugs but also to protect the efficacy of artemisinin where resistance to partner drugs has not arisen. In this respect, new partner drugs with pharmacokinetic profiles closer to those of the artemisinins may be useful. Additionally, combinations of new drugs with longer half-lives may constitute future “single encounter” therapies. Currently, over 50 projects to develop new antimalarial drugs are underway^73^; the next generation of antimalarial compounds is likely to emerge from whole cell screening and medicinal chemistry based on structure-activity relationships. Novel antimalarial drugs and drug combinations will require years of safety and efficacy trials, regulatory reviews and post-marketing surveillance studies before they are widely available for use in human populations. To prevent or delay the future emergence of antimalarial drug resistance, the case for building sustainable approaches for developing these drugs is compelling. To meet these goals, new antimalarial drug regimens are likely to include non-artemisinin drug combinations and may involve triple rather than double combinations, in which the partner drugs should ideally have matching pharmacokinetic and pharmacodynamic properties.

### Evidence of possible spread or emergence of new foci of artemisinin-resistant *P. falciparum*, and current efforts to contain them.

**Figure 1.**
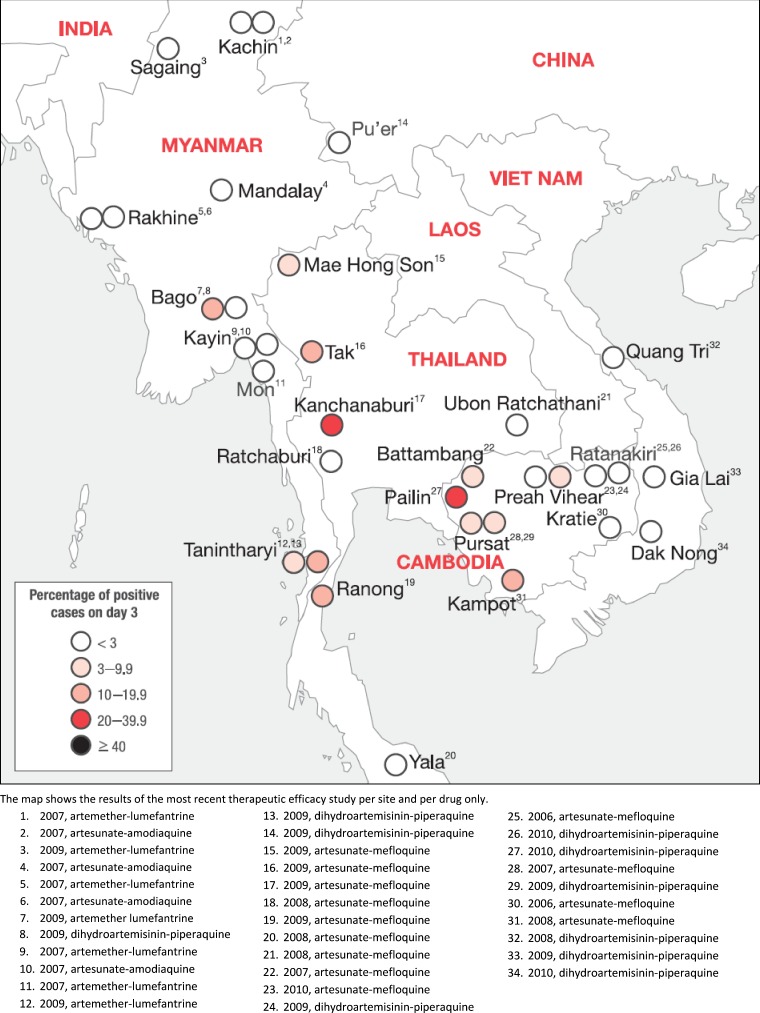
Percentages of patients with *Plasmodium falciparum* parasitemia on Day 3 after treatment with an ACT (2006–2010).

The first well-documented evidence of artemisinin resistance in patients was only recently reported from studies conducted along the Cambodia-Thailand border (Figure 1).^12^,^74^ The true origin and present extent of artemisinin-resistant *P. falciparum* is unknown and studies to map its current distribution are underway.^75^ We do have some indication, however, that parasite responses to artemisinin vary across the Greater Mekong Subregion and may be worsening in some areas. For example, the proportion of patients in Pailin, Cambodia, who were still parasitemic after 3 days of dihydroartemisinin–piperaquine treatment increased from 26% in 2008 to 45% in 2010.^14^ In another study, over 40% of patients in Pailin and Tasanh, Cambodia, were parasitemic after 3 days of artesunate monotherapy.^14^ Recent studies from the Thai-Myanmar border show a significant slowing of parasite clearance rates. A small proportion of the parasite population there has a similar phenotype and degree of heritability of the slow clearance phenotype as that in Western Cambodia.^76^ It is unclear at present whether this represents spread of artemisinin-resistant parasites from the original focus to the Thai-Myanmar border or the emergence of a new focus.

Studies in the Greater Mekong Subregion indicate that the slowing of parasite clearance rates can be attributed in part to changes in parasite genetics.^13^ Although this finding suggests that mutations conferring artemisinin resistance could spread to parasite populations in contiguous geographic areas, this possibility would depend on the fitness cost of the mutations as well as parasite population structure and gene flow. Indeed, we have no information at present to suggest that artemisinin-resistant parasitemia is associated with increased carriage of gametocytes, or whether such gametocytes are transmissible. The Cambodia-Thailand border region previously hosted an early population of chloroquine-resistant *P. falciparum* parasites, which then spread to sub-Saharan Africa; parasite resistance to both sulfadoxine and pyrimethamine also arose in Asia and spread to Africa.^77^,^78^ Because artemisinins are used in combination with effective partner drugs, and their mechanism of action likely differs from those partner drugs, it is difficult to predict whether the spread of artemisinin-resistant *P. falciparum* will follow a similar course.

In collaboration with a wide range of stakeholders, the WHO recently launched the Global Plan for Artemisinin Resistance Containment (GPARC). The GPARC is an ambitious effort to protect ACTs as effective treatments for *P. falciparum* malaria. The four pillars of GPARC are 1) stop the spread of drug-resistant parasites, 2) increase monitoring and surveillance of antimalarial drug resistance, 3) improve access to malaria diagnostic testing and rational treatment with ACTs, and 4) invest in artemisinin-related research. The proposed containment strategies that comprise pillar 1 of GPARC depend strongly on whether artemisinin resistance is confined to this region and has not independently emerged outside of it. The ARCE project, launched in 2008, has targeted its activities to two geographic zones. Zone 1 includes areas where artemisinin-tolerance has already been detected in *P. falciparum*. In Cambodia, these areas comprise the provinces of Pailin, Battambang, Pursat, and Kampot (population 268,000); in Thailand, they comprise select areas in the provinces of Trat (Bo Rai District) and Chanthaburi (Pong Nam Ron and Soi Dao Districts) that border Western Cambodia (population 112,000). Zone 2, defined as the provinces neighboring Zone 1, currently comprise an area supporting a population of 11 million people where the risk of emerging artemisinin resistance runs high because of its proximity to Zone 1.

To begin containing artemisinin-resistant *P. falciparum*, the ARCE has adopted a multidisciplinary effort involving 1) large-scale distribution of long-lasting, insecticide-treated nets; 2) free diagnosis and treatment of malaria at the village level; 3) 24-hour health care facilities to diagnose and treat malaria; 4) intensive post-treatment surveillance for recrudescent parasites in patients treated for malaria; 5) behavior change education programs led by “Village Malaria Workers” to reduce transmission and increase awareness about the importance of taking drugs correctly; 6) innovative means to reach mobile populations; 7) concerted efforts to stop the supply and use of fake and substandard drugs; 8) stringent measures to stop the supply and use of single drug treatments (e.g., artemisinin monotherapy); 9) focused screening and treatment of asymptomatic parasitemic individuals in most malarious border villages using highly sensitive molecular diagnostic methods; 10) basic scientific research to develop new and longer lasting drugs; and 11) operational research to better mobilize resources for containment.^79^ All these efforts have been integrated into an online system that enables the real-time mapping of *P. falciparum*-infected individuals at the village- and, in some cases, house-level.^80^ Since 2009, the incidence of falciparum malaria has decreased in the target zone on the Cambodia-Thailand border, suggesting these containment efforts are having their expected impact.^81^ However, the proportion of patients with *P. falciparum* malaria who are parasitemic on Day 3 after treatment with an ACT has continued to rise, suggesting that only the complete elimination of this pool of parasites is likely to stop the spread of artemisinin-resistant parasites which is also suggested by mathematical modelling.^82^

Although there is no standard formula for malaria elimination, the focused screening and treatment of parasitemic individuals will play an essential role, and the use of interventions other than the standard malaria control measures will certainly be necessary. Many combinations of interventions have been proposed, but testing all of them under field conditions is not feasible. Mathematical modeling can help guide the selection of malaria control and elimination strategies. Models have already informed malaria policies and programs; for instance, the strategy of GPARC to drive malaria incidence down to elimination levels along the Cambodia-Thailand border was informed by a mathematical model as the only way to contain artemisinin-resistant *P. falciparum*.^82^

The use of drugs that kill *P. falciparum* gametocytes can amplify the impact of other interventions by blocking transmission of artemisinin-resistant parasites from humans to mosquitoes. Primaquine, tafenoquine, and other 8-aminoquinolines effectively kill mature gametocytes.^83^ Rapid and effective malaria control in Cambodia through mass administration of artemisinin-piperaquine with low doses of primaquine has been reported to be effective.^84^ However, primaquine, the only licensed transmission-blocking antimalarial drug, causes intravascular hemolysis in patients with glucose-6-phosphate dehydrogenase (G6PD) deficiency. A risk-benefit analysis of the use of primaquine as a mass drug treatment, based on the prevalence of G6PD deficiency in the Greater Mekong Subregion, remains incomplete and is thus a priority for elimination efforts. The occupational migration of human populations along the Cambodia-Thailand border is likely to contribute substantially to the spread of artemisinin resistance. Studies to describe the movement and health-seeking behaviors of these populations are essential to reduce the prevalence of *P. falciparum* infection in this region and are currently underway.

Another issue jeopardizing efforts to contain artemisinin resistance is the high prevalence of substandard and counterfeit antimalarial drugs in Southeast Asia, along with the continued illegal sale of monotherapies.^85^ Counterfeit and substandard antimalarial drugs can contain inadequate levels of active pharmaceutical ingredients.^85^ Exposure to sub-therapeutic levels of drugs can drive the selection of drug-resistant parasite strains.^86^,^87^ To prevent this scenario, the relevant authorities will need to strengthen their efforts at ensuring drug quality, regulating drug supply, and educating communities about the hazards of substandard drugs. Traditionally under-emphasized, the problem of counterfeit and substandard drugs has come to the forefront of international health agendas with the emergence of artemisinin resistance. Collaborations between national drug regulatory authorities, INTERPOL, the WHO's International Medical Products Anti-Counterfeiting Taskforce, the Counterfeit Drug Forensic Investigation Network, and the U.S. Pharmacopeia are actively tackling this issue.

### Resources for the future.

Resources available to manage and control artemisinin resistance include government public health offices, academia, research institutions, policy programs, philanthropic organizations, and the pharmaceutical industry. With strategic guidance from the Global Malaria Program at WHO, governments of affected countries are leading implementation efforts to contain artemisinin resistance. Artemisinin resistance research, control, and elimination activities receive financial contributions from endemic country governments, Bill & Melinda Gates Foundation, U.K. Department for International Development, U.S. National Institutes of Health, U.S. Agency for International Development, Wellcome Trust of Great Britain, Institute Pasteur, and Global Fund to Fight AIDS, Tuberculosis, and Malaria, among others.

Surveillance efforts are led by the national malaria control programs, and the WHO is the entity tracking progress with surveillance of artemisinin resistance worldwide. Available surveillance data are being made publicly available on a global web-based database by the Worldwide Antimalarial Resistance Network (WWARN, http://www.wwarn.org), which has developed a strong networking and data-sharing agenda. More specifically, TRAC aims to map the artemisinin resistance phenotype across Southeast Asia and at sentinel sites in Africa using clinical research and *in vitro* experimental protocols that have been harmonized across sites. This multi-site study is centered in four provinces in Cambodia and includes sites in the neighboring countries of Vietnam, Laos, Thailand, Myanmar, India, and Bangladesh. Numerous groups and institutes are involved in collaborative research efforts to identify molecular markers of artemisinin resistance. WWARN (in collaboration with TRAC) has opened a specimen management center that will collect valuable clinical samples from surveillance studies and clinical trial sites, and disseminate them for studies aiming to identify resistance markers that can serve as tools for surveillance.

The Coordination, Rationalization, and Integration of Antimalarial Drug Discovery and Development Initiatives (CRIMALDDI) coordinates malaria research initiatives in antimalarial drug discovery and development, aligns the European efforts with international initiatives, engages industry, and provides technical guidance on standardization of core requirements of regulatory drug development. Operational research for delivery of subsidized ACTs is being conducted by the Affordable Medicines for Malaria (AFMm) group in partnership with Population Services International. Drug discovery efforts are global, with notable efforts from the Medicines for Malaria Venture as they support the discovery, development, and delivery of new, effective, and affordable antimalarial drugs. Vector control efforts, including large-scale insecticide-treated bed nets and indoor residual spraying, are being carried out by endemic country governments. Modeling efforts to guide containment strategies have increased, and collaboration between groups can harness the synergistic value of different approaches. Because little is known about the mechanism of artemisinin resistance and no estimates exist on related morbidity and mortality if this phenotype spreads, modeling efforts are particularly useful to guide future planning. This is not a comprehensive list of resources; many other players are directly and indirectly supporting containment efforts in the Greater Mekong Subregion. As the risks of this public health problem escalate, the need for a secure common platform to compile sensitive data, harmonize data sets, and build a coordinated global effort to mobilize the malaria community also grows.

### Emergent issues and the way forward.

Emerging artemisinin resistance on the Cambodia-Thailand border and the risks it poses to global efforts to eliminate malaria are widely recognized. A containment strategy spearheaded by WHO/GPARC is in place, but a targeted response will require that many unknowns be addressed first. The research agenda for artemisinin resistance outlines the urgent need for success in conducting *in vivo* parasite clearance rate studies, developing new *ex vivo* and *in vitro* antimalarial drug response assays, discovering molecular markers for resistance, creating rapid tests to measure drug quality, and discovering treatments that kill artemisinin-resistant parasites and block their transmission to mosquitoes (Table 1).

Responding to these needs involves raising awareness, being proactive, screening for early evidence of artemisinin resistance outside Southeast Asia, and building research and implementation networks within and outside the malaria community (Table 2). Challenges exist in establishing high-functioning collaborations, and coordinating and publicizing research, surveillance, and operational efforts. In addition, translation of research findings still suffers a large lag time between initially capturing data in the field and subsequently gaining access to relevant public health information. Identifying information gaps and opportunities for collaboration is imperative. In some instances, the public health value of data sharing is not enough; building incentives for investigators with publication privileges and/or other built-in professional opportunities can help facilitate the transfer of information.

The strength of any network model for collaboration depends on the depth and reach of the partnership (Table 3). The sharing of protocols to standardize the collection and analysis of data and samples is one way to better align the various diverse research efforts addressing artemisinin resistance. Such a partnership can also improve the quality control and quality assurance of research methods to avoid false alarms of artemisinin resistance. Finally, advocacy efforts to improve awareness and funding are needed to support the long- and short-term goals of malaria elimination so that this vital enterprise can continue unabated.

## Figures and Tables

**Table 1:** Summary of genetic polymorphisms that are candidate molecular markers for *P. falciparum* resistance to artemisinins or ACT partner drugs

*Gene* ID	Chromosome	Protein type	Polymorphism	Associated with resistance to:	References
*pfcrt* MAL7P1.27	7	Transmembrane transporter	K76T most important 72, 74, 75, variant > 6 other SNPs Splicing variants?	Chloroquine Amodiaquine Quinine Lumefantrine Artemisinins	36–41
*pfmdr1* PFE1150w	5	P-glycoprotein homologue (ABC transporter)	5 SNPs (86, 184, 1034, 1042, 1246) Gene amplification	Chloroquine Amodiaquine Mefloquine Lumefantrine Quinine Artemisinins	42–47
*pfatp6* PFA0310c	1	Sarco/endoplasmic reticulum calcium-dependent (SERCA) ATPase	2 SNPs L263E (engineered) S769N (found in field samples in French Guiana)	Artemisinins	41
*G7* PF13_0271	13	ABC transporter	Trinucleotide insertion (found in Thailand)	Artemisinins	48,49
*pfmrp1* PFA0590w	1	ABC transporter	SNPs	Artemisinins Lumefantrine Sulphadoxine-pyrimethamine	50,47
*pfubp1* PFA0220w	1	Ubiquitin carboxyl terminal hydrolase	SNPs, indels	Artemisinins (in *P. chabaudi*)	51
*pfap2-mu* PFL0885w	12	Clathrin adaptor protein mu subunit	SNPs	Artemisinins (in *P. chabaudi*)	(Hunt and others, unpublished data)

**Table 2:** Research needs to address artemisinin resistance

1. Discover the mechanisms of artemisinin drug action and parasite resistance to artemisinins.
2. Conduct *in vivo* parasite clearance rate studies for monitoring and surveillance of the phenotype.
3. Develop new *ex vivo* and *in vitro* antimalarial drug response assays.
4. Discover molecular markers for resistance.
5. Create low-cost rapid tests to measure drug quality.
6. Discover treatments that kill artemisinin-resistant parasites and block their transmission to mosquitoes.
7. Discover new partner drugs for combination with artemisinins to treat malaria.
8. Build research models to estimate the impact of artemisinin resistance spreading to other endemic regions, to help plan emergency preparedness strategies.

**Table 3:** Implementation needs to address artemisinin resistance

1 Create, troubleshoot, and sustain high-functioning collaborations that bridge coordination and publicizing of research, surveillance, and operational efforts.
2. Reduce the lag time between translations of relevant laboratory research to targeted public health changes.
3. Share protocols to standardize the collection and analysis of data and samples to better align the various diverse research efforts addressing artemisinin resistance.
4. Advocate efforts and strategies to improve awareness and funding allocation that support goals of malaria elimination.
